# Retraction of ‘Functional dissection of the C‐terminal domain of type II DNA topoisomerase from the kinetoplastid hemoflagellate *Leishmania donovani*’

**DOI:** 10.1093/nar/gkab317

**Published:** 2021-05-07

**Authors:** Tanushri Sengupta, Mandira Mukherjee, Chhabinath Mandal, Aditi Das, Hemanta K Majumder

**Affiliations:** Department of Molecular Parasitology, Department of Drug Design, Development and Molecular Modeling, Indian Institute of Chemical Biology, Kolkata 700032, India; Department of Molecular Parasitology, Department of Drug Design, Development and Molecular Modeling, Indian Institute of Chemical Biology, Kolkata 700032, India; Department of Drug Design, Development and Molecular Modeling, Indian Institute of Chemical Biology, Kolkata 700032, India; Sealy Center for Molecular Sciences, University of Texas Medical Branch at Galveston, Galveston, TX 77555, USA; Department of Molecular Parasitology, Department of Drug Design, Development and Molecular Modeling, Indian Institute of Chemical Biology, Kolkata 700032, India

*Nucleic Acids Research*, Volume 31, Issue 18, 15 September 2003, Pages 5305–5316, https://doi.org/10.1093/nar/gkg727

In August 2020, the Editors were alerted by a reader that the bacterial colonies depicted in Figure 2B of the above article had been duplicated within and across the figure panels despite using different clones and incubation conditions (1). In Figure 3C, multiple lanes have also clearly been spliced in.

Details of the duplications and splicing have been published in PubPeer: https://pubpeer.com/publications/34E9446142CBE8FCD55311F1EB9C1C

The Authors’ institution (Indian Institute of Chemical Biology) had already investigated in 2019 and concluded that there was strong evidence of image manipulation. They also recommended that the raw data should be examined. However, the Authors are unable to provide the original data or a reasonable explanation for the image manipulations.

The Editors and Authors are therefore jointly retracting the article from *Nucleic Acids Research*.
